# Online/Offline MA-CP-ABE with Cryptographic Reverse Firewalls for IoT

**DOI:** 10.3390/e25040616

**Published:** 2023-04-04

**Authors:** Juyan Li, Ye Fan, Xuefen Bian, Qi Yuan

**Affiliations:** 1 College of Data Science and Technology, Heilongjiang University, Harbin 150080, China; lijuyan@hlju.edu.cn (J.L.); fy180253@163.com (Y.F.); 2 College of Telecommunication and Electronic Engineering, Qiqihar University, Qiqihar 161006, China; foreverquanqi@126.com

**Keywords:** MA-CP-ABE, CRF, online/offline, outsourced decryption, IoT

## Abstract

Devices in the Internet of Things (IoT) usually use cloud storage and cloud computing to save storage and computing cost. Therefore, the efficient realization of one-to-many communication of data on the premise of ensuring the security of cloud storage data is a challenge. Ciphertext-Policy Attribute-Based Encryption (CP-ABE) can not only protect the security of data in the cloud and achieve one-to-many communication but also achieve fine-grained access control for data. However, the single-authority CP-ABE faces the crisis of single point of failure. In order to improve security, the Multi-Authority CP-ABE (MA-CP-ABE) is adopted. Although there are provably-secure MA-CP-ABE schemes, Edward Snowden’s research shows that provably-secure cryptographic schemes are vulnerable to backdoor attacks, resulting in secret disclosure, and thus threatening security. In addition, ABE requires huge computational overhead in key generation, encryption and decryption, which increase with the increase in the number of attributes and the complexity of the access structure, and there are a large number of resource-constrained devices in the IoT. To mitigate this issue, we construct the Online/Offline MA-CP-ABE with Cryptographic Reverse Firewalls (OO-MA-CP-ABE-CRFs) scheme. This scheme not only uses Cryptographic Reverse Firewall (CRF) to resist backdoor attacks but also uses online/offline key generation, online/offline encryption and outsourcing encryption technology to optimize the efficiency of the MA-CP-ABE scheme with reverse firewall, reducing the storage and computing cost of users. Finally, the security of the OO-MA-CP-ABE-CRFs scheme is proved, and the experimental results indicate that the scheme is efficient and practical.

## 1. Introduction

With the increasing number of terminal devices connected to the Internet of Things (IoT), the data to be processed increase exponentially. At the same time, with the widespread use of cloud computing and cloud storage technologies, the data from IoT devices were uploaded to the cloud for storage and processing. Considering that the dishonest cloud seriously threatens the privacy and security of data, it is necessary to encrypt the data before cloud storage. Attribute-Based Encryption (ABE) can not only protect data privacy but also realize fine-grained access control to data.

ABE was originally proposed by Sahai and Waters, which can be classified as Key-Policy ABE (KP-ABE) and Ciphertext-Policy ABE (CP-ABE), respectively. In the CP-ABE (KP-ABE) scheme, the user’s private key (ciphertext) is associated with its attributes, and the ciphertext (private key) is associated with the access structure. Only when the attributes of the user meet the access structure of the ciphertext can the user use the private key to recover the plaintext correctly. Because users can use CP-ABE to specify flexible access structures for ciphertext, they can achieve fine-grained access control for ciphertext, so CP-ABE has been widely used in cloud computing [1,2,3,4,5,6]. Because the user’s private key is generated by an attribute authority, the attribute authority knows the user’s private key and can decrypt the ciphertext. Therefore, the attribute authority is required to be a trusted third party. In addition, the single-authority ABE scheme has a series of negative effects such as heavy computing burden, single point of failure crisis, and excessive central authority. The Multi-Authority ABE (MA-ABE) scheme can solve the problem of limited computing resources and single point of failure in the single-authority ABE scheme, so it has been widely used in the IoT [7,8,9].

Edward Snowden’s disclosure showed that even if the user’s machine is executing the cryptographic scheme normally, it may be placed on the machine by the adversary with undetectable backdoor vulnerabilities, so the user’s secret is exposed, thus endangering the user’s security [10,11,12]. Even provably secure cryptographic schemes face this threat, including the provably secure MA-ABE scheme. The Cryptographic Reverse Firewall (CRF) uses a trusted machine placed between the user’s machine and the external environment to intercept illegal data streams entering the machine and modifying system parameters in time, which can resist internal vulnerabilities such as backdoors, thereby providing users with privacy security [13]. Therefore, it is necessary to construct an MA-ABE scheme with CRFs (MA-ABE-CRFs).

One of the main drawbacks of the ABE encryption scheme is that the computational cost of encryption, decryption and key generation increases with the increase in the complexity of the access structure or the number of attributes. MA-ABE-CRFs faces the same drawback because of the increase in attribute authorities and the increase in CRFs, which lead to higher computing cost. The devices in the IoT have limited storage and computing resources, so it is necessary not to use MA-ABE directly. The online/offline key generation, online/offline encryption and outsourced decryption to improve the efficiency of MA-ABE are considered [14]. The online/offline technology splits the computation for MA-ABE into two phases: an offline preparation phase, in which users can do a lot of work, such as offline key generation and offline encryption; and a second phase, where once the user knows the encrypted plaintext and attributes/access structure, the user can quickly generate a key online to encrypt the plaintext. Outsourcing pre-decryption can transfer a large amount of decryption computing cost to cloud service providers, thus reducing users’ decryption computing cost, and is more suitable for resource-constrained IOT devices. Considering the efficiency of MA-ABE-CRFs, especially for IoT devices with limited storage and computing resources, we can consider online/offline key generation, online/offline encryption and outsourced decryption to improve the efficiency of MA-ABE-CRFs [14]. Therefore, it is necessary to study the Online/Offline MA-CP-ABE-CRFs (OO-MA-CP-ABE-CRFs) to ensure the security of data in the IoT and improve the efficiency of data transmission.

In this paper, we combine CRF and CP-ABE, and adopt multi-authority, online/offline, outsourced decryption to construct OO-MA-CP-ABE-CRFs for IoT. The specific contributions are as follows:

(1) We propose a new MA-CP-ABE-CRF scheme, which not only avoids the crisis of single point of failure of single-authority ABE but also provides flexible access control for ciphertext data. In addition, four CRFs are used to re-randomize key parameters. This allows the MA-CP-ABE scheme to maintain functionality and resist ex-filtration even if it is compromised by unexpected attacks.
(2) In order to make the scheme suitable for IoT, we have adopted online/offline key generation, online/offline encryption, and outsourcing decryption technologies to improve the computational efficiency of the scheme. These technologies are not only adopted by users and attribute authority but also by the four CRFs, which can significantly improve the efficiency of the scheme. Compared with other studies in terms of computational and storage overhead, our scheme has obvious advantages.
(3) We have theoretically analyzed and proven the correctness and security of the OO-MA-CP-ABE-CRFs scheme, including CPA security, weak security reservation, and weak demonstration resistance. These security guarantees that devices in IOT are secure even when attacked by backdoors.

The rest of this article is organized as follows. Section 2 discusses the related work. Section 3 presents the preliminaries. Section 4 describes the proposed OO-MA-CP-ABE-CRFs scheme. Section 5 presents the performance analysis of the proposed scheme. Section 6 presents a real-world application of the proposed scheme. Finally, Section 7 concludes this work.

## 2. Related Work

This section mainly summarizes the related works on ABE, CRF and online/offline cryptography.

### 2.1. Attribute-Based Encryption

ABE was originally proposed by Sahai and Waters on the basis of Fuzzy Identity-Based Encryption (FIBE) [15]. Goyal et al. [16] extended FIBE technology to ABE technology and defined two types of ABE, called KP-ABE and CP-ABE, respectively. The complete framework of the first anti-collusion CP-ABE scheme was proposed by Bethencourt et al. [17]. Since CP-ABE can customize access control policies by data owners, it is widely used in various cloud scenarios due to the advantages of fine-grained access control. Because the single-authority ABE scheme has a series of negative effects such as heavy computing burden, single point of failure crisis, and excessive central authority, Chase [18] constructed an MA-ABE scheme to solve such problems. Chase et al. [19] further protect user privacy by eliminating the trusted central authority to prevent information from being concentrated on specific users. Lin et al. [20] proposed a threshold-based multi-authority FIBE scheme that can be extended to MA-ABE. Qian et al. [21] proposed an MA-ABE scheme supporting attribute revocation and dynamic policy updates to meet the privacy security requirements of patients in the personal health record (PHR) system.

### 2.2. Cryptographic Reverse Firewall

Due to the influence of Snowden’s ex-filtration incident, Mironov et al. [13] introduced the concept of a CRF, which aims to intercept and update the receiving and sending messages of the client in time to prevent malicious adversary in the system. Zhou et al. [22] proposed an IBE-based CRF scheme. Chen et al. [23] rely on the malleable smooth projective hash function with key malleability and element re-randomizability to construct multiple CRF-based cryptographic protocols. Zhou et al. [24] designed a single-round CL-PKE-CRF protocol with low communication overhead. Zhou et al. [25] proposed a searchable public key encryption scheme based on CRF, which can resist various attack methods without a secure channel, thereby fully guaranteeing the user’s information security. Ma et al. [26] designed an online/offline CP-ABE-CRF scheme, which effectively reduces the computational overhead while resisting secret ex-filtration, and further ensures the practicability of the scheme on lightweight devices. Hong et al. [27] constructed an MA-KP-ABE-CRF scheme, which supports a non-monotonic access structure.

### 2.3. Online/Offline Cryptography

It is also worth noting that not only does the CRF framework bring a lot of computing overhead, but most of the ABE scheme design process itself generally has expensive computing operations. Many researchers use online/offline technology to solve this problem. Khan et al. [28] entrusted the heaviest computing operations to the offline stage through online/offline technology, which reduced computing overhead, and designed an online/offline-aided attribute-based multi-keyword search scheme. In order to reduce the communication overhead in the verification phase, Ali et al. [29] designed a verifiable online/offline multi-keyword search scheme. For fields such as smart grids with high security and timeliness, Zhang et al. [30] outsourced a large number of calculations to the encryption and decryption server, reducing the calculation overhead of the client, and constructed outsourcing attributed-based ranked searchable encryption. In order to further reduce the computational burden of data owners and clients, Shao et al. [31] based on the online/offline MA-ABE scheme combined with key conversion technology to outsource the complex operation of the decryption stage to the proxy server.

## 3. Preliminaries

This introduces preliminaries of the OO-MA-CP-ABE-CRFs scheme.

### 3.1. Bilinear Groups

Let G, $G_{T}$ be two multiplicative cyclic groups with a prime order of *p*. Among them, *g* is the generator of the group G, and the bilinear map $e:G\times G\to G_{T}$ has the following three properties:

(1) Bilinearity: For any P,Q∈G and $a,b\in Z_{p}^{*}$, it can calculate; $e\left(P^{a},Q^{b}\right)=e{\left(P,Q\right)}^{ab}$.
(2) Non-degeneracy: If P,Q∈G is assumed, then e(P,Q)≠1 is established;
(3) Computability: For any P,Q∈G, there exists an efficient algorithm to compute e(P,Q).

q−type assumption. The challenger first calls parameters of bilinear pairings *p*, G, $G_{T}$, *e*, and picks a random group element g∈G and random exponents $a,s,b_{1},b_{2},\ldots ,b_{q}\leftarrow Z_{q}$. Then, the challenger sends the following terms to the adversary.

$g,g^{s}$

$g^{a^{i}},g^{b_{j}},g^{a^{i}/b_{j}^{2}},\forall \left(i,j\right)\in \left[q,q\right]$

$g^{a^{i}/b_{j}},\forall \left(i,j\right)\in \left[2q,q\right]withi\neq q+1$

$g^{a^{i}b_{j}/b_{j^{\prime }}^{2}},\forall \left(i,j,j^{\prime }\right)\in \left[2q,q,q\right]withj\neq j^{\prime }$

$g^{sa^{i}b_{j}/b_{j^{\prime }}},g^{sa^{i}b_{j}/b_{j^{\prime }}^{2}},\forall \left(i,j,j^{\prime }\right)\in \left[q,q,q\right]withj\neq j^{\prime }$

Finally, the challenger flips a random coin b∈{0,1}, sets $T=e{\left(g,g\right)}^{sa^{q+1}}$ if b=0 and otherwise $T\in G_{T}$ is a random term, and sends *T* to attacker. The attacker outputs a guess $b^{\prime }\in \left\{0,1\right\}$ for *b*. The advantage of the attacker in the game is $|\mathrm{Pr}\left[b=b^{\prime }\right]-\frac{1}{2}|$. We say that the q−type assumption holds if all probabilistic polynomial time attackers have at most a negligible advantage in the above security game.

### 3.2. Linear Secret Sharing Schemes

Assuming that $\left\{P_{1},P_{2},\ldots ,P_{n}\right\}$ is a set of participants, if B∈A and B⊆C have C∈A, then we say that the set $A\subseteq 2^{\left\{P_{1},P_{2},\ldots ,P_{n}\right\}}$ is monotonous for any *B* and *C*. A monotonic access structure is the set of all non-empty subsets of $\left\{P_{1},P_{2},\ldots ,P_{n}\right\}$, namely $A\subseteq 2^{\left\{P_{1},P_{2},\ldots ,P_{n}\right\}}\backslash \left\{⌀\right\}$. For this reason, sets in A are called authorized sets, whereas sets not in A are called non-authorized sets.

A linear secret sharing scheme Π with a set of parties is linear on $Z_{p}$, the following conditions need to be satisfied:

(1) The shares of each party constitute a vector over $Z_{p}$;
(2) There exists a share-generating matrix *M* with *l* rows and *n* columns for scheme Π. Furthermore, there exists a function ρ that maps each row of the matrix *M* to an associated party. For example, each row i∈[l] of the matrix is closely related to ρ(i), where [l]=1,…,l. For column vector $\vec{v}=\left(s,r_{2},\ldots ,r_{n}\right)$, we choose *s* from $Z_{p}$ as the secret value that needs to be shared, and $r_{2},\ldots ,r_{n}\in Z_{p}$ are randomly selected. $M\vec{v}$ represents a vector composed of *l* elements, and each element is the secret share generated by the scheme Π for *s*. The share ${\left(M\vec{v}\right)}_{i}$ belongs to party ρ(i).

### 3.3. Cryptographic Reverse Firewall

The CRF was originally proposed by Mironov and Stephens Davidowitz to provide a strong security backing for modern cryptographic algorithms to avoid the threat of backdoors. CRF acts as a function of message interception and parameter update in the entire cryptographic system. Deploying a cryptographic scheme that correctly implements CRF can effectively ensure that the scheme can still retain its security even if it runs on an infected machine. Appendix A provides more details about CRF.

### 3.4. System Model

**Figure 1:**
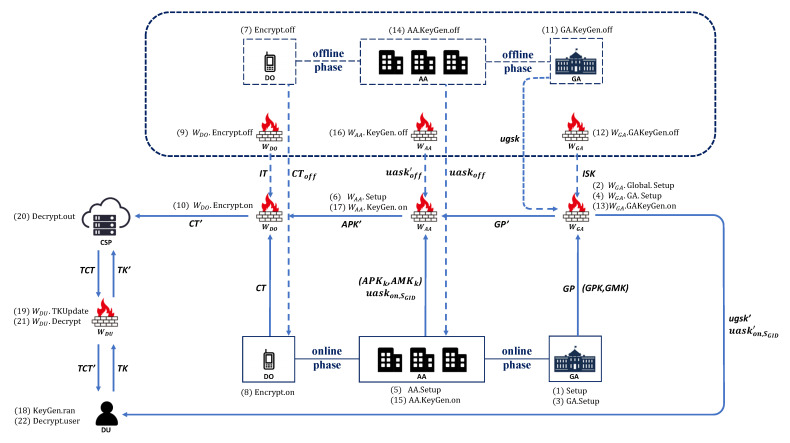
System architecture of OO-MA-CP-ABE-CRFs.

As shown in Figure 1, the scheme includes five participants and corresponding reverse firewalls. They are the global identity authority (GA), attribute authorities (AA), the cloud service provider (CSP), the data owner (DO) and the data user (DU). Among them, the CRF of GA is $W_{\mathrm{GA}}$, the CRF of AA is $W_{\mathrm{AA}}$, the CRF of DO is $W_{\mathrm{DO}}$, and the CRF of DU is $W_{\mathrm{DU}}$.

Specifically, (1) GA generates global parameter GP. (2) If the process is corrupted, then $W_{\mathrm{GA}}$ randomizes the GP, obtains and broadcasts $GP^{\prime }$ globally. (3) GA outputs its own public/private key pair (GPK,GMK) by $GP^{\prime }$. (4) If the process is compromised, $W_{\mathrm{GA}}$ outputs the updated public/private key pair $\left(GPK^{\prime },GMK^{\prime }\right)$, and returns it to GA. (5) AA outputs its own public/private key pair $(APK_{k},AMK_{k})$. (6) If the process is compromised, $W_{\mathrm{AA}}$ ouputs the updated public/private key pair $AP{K_{k}}^{\prime }$, $AM{K_{k}}^{\prime }$. (7) DO offline encryption and output offline ciphertext ${CT}_{off}$. (8) DO encrypts plaintext online and outputs the ciphertext $CT_{A}$ under the access structure A. (9) If the encryption process is compromised, $W_{\mathrm{DO}}$ outputs the updated intermediate ciphertext IT offline. (10) $W_{\mathrm{DO}}$ outputs the updated ciphertext $CT^{\prime }$. (11) GA outputs the user’s decryption key ugsk offline. (12) If the process is compromised, $W_{\mathrm{GA}}$ outputs the intermediate secret key ISK offline. (13) $W_{\mathrm{GA}}$ outputs the user’s updated decryption key $ugsk^{\prime }$ online. (14) AA outputs the user’s offline decryption key ${uask}_{off}$. (15) AA outputs the decryption key ${uask}_{on,S_{GID}}$ of the user online. (16) If the process is compromised, $W_{\mathrm{AA}}$ of ${AA}_{k}$ outputs the updated offline decryption key $uas{k_{off}}^{\prime }$.(17) $W_{\mathrm{AA}}$ outputs the user’s updated online decryption key $uask_{on,S_{GID}}^{\prime }$ online. (18) In order to realize outsourcing pre-decryption, DU outputs a conversion key TK and a retrieval key RK. (19) If the process is compromised, $W_{\mathrm{DU}}$ outputs an updated conversion key $TK^{\prime }$ and a corresponding random β. (20) CSP performs outsourcing pre-decryption and outputs the transformed ciphertext TCT. (21) If the process is compromised, $W_{\mathrm{DU}}$ outputs an updated transformed ciphertext $TCT^{\prime }$. (22) DU outputs the plaintext. For the normalized definition of OO-MA-CP-ABE-CRF, see Appendix B.

### 3.5. Security Model

We define the security model of the OO-MA-CP-ABE-CRFs scheme based on the security model [26,27]. Similar to [26,27], it is assumed that GA, AA, DO and DC are fully trusted, and the CSP is semi-trusted. Because the algorithms (Global.Setup, GASetup, AASetup, GA.KeyGen.off, GA.KeyGen.on, AA.KeyGen.off, AA.KeyGen.on, Encrypt.off, Encrypt.on and KeyGen.ran) in the scheme still maintain functionality after implanting malicious trapdoors, so it is necessary to consider that these algorithms may be compromised without the knowledge of the executor. In addition, considering that $W_{\mathrm{DO}}$ and $W_{\mathrm{DU}}$ are curious about the user’s data, we assume that $W_{\mathrm{DO}}$ and $W_{\mathrm{DU}}$ are semi-trusted. Since $W_{\mathrm{GA}}$, $W_{\mathrm{AA}}$ have access to the decryption key of the user, it is assumed that $W_{\mathrm{GA}}$, $W_{\mathrm{AA}}$ are completely trusted. Additionally, all CRFs are considered trusted zones and cannot be tampered with by any outsiders.

The CPA security game for OO-MA-CP-ABE-CRFs is played by a challenger C and an adversary A.

**Initialization**: Adversary A sends the access structure $A^{*}$ and the functionality maintaining algorithms to challenger C.

**Setup**: The Setup algorithm is executed by challenger C. The updated global public parameter $GP^{\prime }$, updated public key $GPK^{\prime }$ and $AP{K_{k}}^{\prime }$ of the authority are sent to the adversary A.

**Phase 1**: The adversary A can adaptively query the Key Generation Oracle (KGO). A queries for attribute set $S_{i}$ and user identity GID, which requires that $S_{i}$ does not satisfy the challenge policy $A^{*}$, i=1,2,⋯,q. The challenge C replies to adversary with user’s updated decryption key $\left(ugsk^{\prime },uask_{S_{GID}}^{\prime }\right)$, and updated conversion key $TK^{\prime }$.

**Challenge**: Adversary A sends two equal-length plaintexts $m_{0}$, $m_{1}$ to C. C selects a random bit b∈{0,1}, and sends the updated ciphertext $CT_{b}^{\prime }$ to the adversary A, where $CT_{b}^{\prime }$ is the ciphertext of $m_{b}$ under access structure $A^{*}$.

**Phase 2**: The process is the same as Phase 1.

**Guess**: The adversary A outputs a guess $b^{\prime }\in \left\{0,1\right\}$ for *b*. The advantage of A in the game is $|\mathrm{Pr}\left[b=b^{\prime }\right]-\frac{1}{2}|$.

**Definition**: The OO-MA-CP-ABE-CRFs is CPA secure if all probabilistic polynomial time (PPT) adversaries have at most a negligible advantage in the above security game.

## 4. OO-MA-CP-ABE-CRFs

In this section, we first construct a basic OO-MA-CP-ABE scheme based on [32]. The random of the ciphertext and key in this scheme is all re-randomized. Then, based on this basic OO-MA-CP-ABE scheme, we construct an OO-MA-CP-ABE-CRFs scheme, and finally, prove the security of the constructed scheme.

### 4.1. Basic Construction of OO-MA-CP-ABE Scheme

In order to make the basic OO-MA-CP-ABE scheme suitable for constructing the CRF framework, we construct a concessive OO-MA-CP-ABE scheme, which can be divided into four phases and contains a total of 11 algorithms.

(1) **Initialization phase**. GA and AA perform initialization.

➀ Global.Setup(λ,U)→GP. GA chooses a security parameter λ and describes a tuple $\left(G,G_{T},p,e\right)$, where G and $G_{T}$ are two cyclic multiplicative groups of large prime order *p* and $e:\left(G\times G\right)\to G_{T}$ is a bilinear map. Let *g* be a generator of G. GA randomly chooses h,u,v,w←G and outputs the global system parameters GP=(g,h,u,v,w).

➁ $\mathrm{GA}.\mathrm{Setup}(GP)\to \left(GPK,GMK\right)$. GA randomly chooses α←Z, outputs $GPK=e{\left(g,g\right)}^{\alpha }$, and keeps GMK=α by itself.

➂ $\mathrm{AA}.\mathrm{Setup}(GP,k,U_{k})\to (APK_{k},AMK_{k})$. It first takes $GP,k,U_{k}$ as input, where k∈[K]. Then, $AA_{k}$ randomly chooses $\alpha_{k}\leftarrow Z_{p}$, and lets $\left(\hat{u},\hat{h}\right)=\left(u^{\alpha_{k}},h^{\alpha_{k}}\right)$. Finally, it outputs $APK_{k}=\left(\hat{u},\hat{h}\right)$ and keeps $AMK_{k}=\alpha_{k}$.

(2) **Encryption phase**. DO encrypts plaintext offline and online.

➃ $\mathrm{Encrypt}.\mathrm{off}(GP,GPK,APK)\to CT_{off}$. The algorithm takes $GP,GPK,APK=\cup_{k\in [K]}APK_{k}$ as input, and the DO randomly chooses $s\leftarrow Z_{p}$, $t_{j}\leftarrow Z_{p}$, j∈[J], *J* is used by DO to determine the size of the offline ciphertext pool. It calculates $Km=GPK^{s}=e{\left(g,g\right)}^{\alpha s}$, $C_{0}^{\prime }=g^{s}$, ${C_{j,1}}^{\prime }={\left(\hat{h}\right)}^{-t_{j}}$, ${C_{j,2}}^{\prime }=g^{t_{j}}$, ${C_{j,3}}^{\prime }=v^{t_{j}}$, and outputs $CT_{off}=\left(s,Km,{C_{0}}^{\prime },{\left\{{C_{j,1}}^{\prime },{C_{j,2}}^{\prime },{C_{j,3}}^{\prime }\right\}}_{j\in [J]}\right)$.

➄ $\mathrm{Encrypt}.\mathrm{on}(GP,APK,m,A,CT_{off})\to CT_{A}$. On input public parameters PK, an intermediate ciphertext IT, a plaintext *m* and an LSSS access structure A=(M,ρ), where *M* is an $l\times n(l\leq N^{\prime })$ matrix. DO randomly chooses $y_{2},\ldots ,y_{n}\leftarrow Z_{p}$, sets $\vec{y}={\left(s,y_{2},\cdots ,y_{n}\right)}^{T}$, and obtains $\vec{\lambda }={\left(\lambda_{1},\lambda_{2},\cdots ,\lambda_{l}\right)}^{T}=M\vec{y}$. In addition, for $j\in \left[l\right]$, suppose $\rho \left(j\right)$ corresponds to an attribute controlled by $AA_{k}$. DO sets C=m·Km, $C_{0}={C_{0}}^{\prime }$, $C_{j,1}={C_{j,1}}^{\prime }{\hat{u}}^{-\rho \left(j\right)t_{j}}={\left(\hat{h}{\hat{u}}^{\rho (j)}\right)}^{-t_{j}}$, $C_{j,2}={C_{j,2}}^{\prime }=g^{t_{j}}$, $C_{j,3}={C_{j,3}}^{\prime }\cdot w^{\lambda_{j}}=w^{\lambda_{j}}v^{t_{j}}$, outputs $CT_{A}=(C,C_{0},\left\{C_{j,1},C_{j,2},C_{j,3}{\}}_{j\in [l]}\right)$.

(3) **Key generation phase**. GA and AA generate decryption keys offline and online for the user.

➅ GA.KeyGen.off(GP,GMK)→ugsk. GA randomly chooses $r\leftarrow Z_{p}$, calculates $K_{0}=g^{\alpha }w^{r}$, $K_{3}=g^{r}$, $D=v^{-r}$, and outputs $ugsk=(K_{0},K_{3},D)$.

➆ $\mathrm{AA}.\mathrm{KeyGen}.\mathrm{off}\left(GP,AMK_{k}\right)\to uask_{off}$. For k∈[K], $AA_{k}$ randomly chooses $r_{i}\leftarrow Z_{p}$, $i\in [|U_{k}|]$, calculates ${K_{i,1}}^{\prime }=g^{\frac{r_{i}}{\alpha_{k}}}$ and ${K_{i,2}}^{\prime }=h^{r_{i}}$, and outputs $uask_{off}={\left\{r_{i},K_{i,1}^{\prime },K_{i,2}^{\prime }\right\}}_{i\in [|U_{k}|]}$.

➇ $\mathrm{AA}.\mathrm{KeyGen}.\mathrm{on}\left(GP,GID,APK_{k},S_{GID,k},uask_{off}\right)\to uask_{on,S_{GID}}$. For $i\in \left[\left|S_{GID,k}\right|\right]$, $AA_{k}$ calculates $K_{i,1}={K_{i,1}}^{\prime }=g^{\frac{r_{i}}{\alpha_{k}}}$, $K_{i,2}={K_{i,2}}^{\prime }\cdot u^{attr_{i}r_{i}}={\left(u^{attr_{i}}h\right)}^{r_{i}}$, it outputs $uask_{on}={\left\{K_{i,1},K_{i,2}\right\}}_{i\in \left[\left|S_{GID,k}\right|\right]}$. It should be noted that the decryption key of the user GID is $\left(ugsk,uask_{on}\right)$.

(4) **Decryption phase**. For outsourcing decryption, DU generates a conversion key and a retrieval key. CSP performs outsourcing decryption by conversion key, and DU performs final decryption by retrieval key.

➈ $\mathrm{KeyGen}.\mathrm{ran}\left(ugsk,uask_{on}\right)\to \left(TK,RK\right)$. The data user DU randomly chooses $\tau \leftarrow Z_{p}$, calculates ${\tilde{K}}_{0}={K_{0}}^{\prime }{}^{\frac{1}{\tau }}=g^{\frac{\alpha }{\tau }}w^{\frac{r}{\tau }}$, ${\tilde{K}}_{3}={K_{3}}^{\prime }{}^{\frac{1}{\tau }}=g^{\frac{r}{\tau }}$ and $\tilde{D}={D^{\prime }}^{\frac{1}{\tau }}=v^{-\frac{r}{\tau }}$. For $i\in \left[\left|S_{GID,k}\right|\right]$, it calculates ${\tilde{K}}_{i,1}={K_{i,1}}^{\prime }{}^{\frac{1}{\tau }}=g^{\frac{r_{i}}{\alpha_{k}\tau }}$, ${\tilde{K}}_{i,2}={{K_{i,2}}^{\prime }}^{\frac{1}{\tau }}={\left(u^{attr_{i}}h\right)}^{\frac{r_{i}}{\tau }}$, finally outputs a conversion key $TK=(S_{GID},{\tilde{K}}_{0},{\tilde{K}}_{3},\tilde{D},{\left\{{\tilde{K}}_{i,1},{\tilde{K}}_{i,2}\right\}}_{i\in \left[\left|S_{GID,k}\right|\right]})$ and a retrieval key RK=τ.

➉ Decrypt.out(TK,CT)→TCT. On input, a conversion key TK for the attribute set $S_{\mathrm{GID}}$ and a ciphertext CT for access structure A. The CSP judges whether $S_{\mathrm{GID}}$ satisfies A, if not, then it returns ⊥. Otherwise, CSP calculates ${\left\{w_{i}\in Z_{p}\right\}}_{j\in I}$ satisfying $\sum_{j\in I}w_{i}{\vec{M}}_{j}=\left(1,0,\cdots ,0\right)$, where $I=\{j|\rho \left(j\right)\in S_{GID}\}\subseteq \left[l\right]$, and ${\vec{M}}_{j}$ is row *j* of matrix *M*. Then, it calculates $B=\frac{e({\tilde{K}}_{0},C_{0})}{\prod_{j\in I}{T_{j}}^{w_{j}}}=e{\left(g,g\right)}^{\frac{\alpha s}{\tau }}$, where $T_{j}=e\left({\tilde{K}}_{i,1},C_{j,1}\right)\cdot e\left({\tilde{K}}_{i,2}\cdot \tilde{D},C_{j,2}\right)\cdot e\left({\tilde{K}}_{3},C_{j,3}\right)$, and outputs decrypted transformed ciphertext TCT=(C,B).

⑪ Decrypt.user(RK,TCT)→m. The algorithm is executed by DU, inputs a retrieval key RK and the transformed ciphertext TCT, and finally decrypts $\frac{C}{{\left(B\right)}^{\tau }}=\frac{e{\left(g,g\right)}^{\alpha s}\cdot m}{{\left(e\left(g,g^{\frac{\alpha s}{\tau }}\right)\right)}^{\tau }}$ to obtain the plaintext *m*.

**Theorem 1.**  *The basic OO-MA-CP-ABE scheme is CPA secure if the OO-MA-CP-ABE scheme in [32] is selective CPA-secure.*

**Proof.**  The form of user key SK and ciphertext CT in the scheme is the same as that in [32]. Therefore, the modification does not affect the security proof. Furthermore, the key-blinding technique in [33] is used. The proof is similar to [33], so it is omitted. □

### 4.2. Construction of OO-MA-CP-ABE-CRFs

We propose the OO-MA-CP-ABE-CRFs based on the above basic construction, which can resist the exfiltration of secret information from arbitrarily compromised functional-maintaining algorithms executed by the GA, AA, DO and DU. The structure of OO-MA-CP-ABE-CRFs is specifically as follows.

(1) **Initialization phase**. GA, AA, $W_{\mathrm{GA}}$ and $W_{\mathrm{GA}}$ perform initialization.

➀ Before broadcasting GP←Setup(˘,U) to other participants, GA first sends GP to $W_{\mathrm{GA}}$ for the algorithm $W_{\mathrm{GA}}$. Global.Setup.

$W_{\mathrm{GA}}$.Global.$\mathrm{Setup}(GP)\to GP^{\prime }$. After receiving GP, $W_{\mathrm{GA}}$ randomly selects $a,b,c,d,e\leftarrow Z_{p}$ and calculates $g^{\prime }=g^{a}$, $u^{\prime }=u^{b}$, $h^{\prime }=h^{c}$, $w^{\prime }=w^{d}$, $v^{\prime }=v^{e}$. The algorithm outputs $GP^{\prime }=\left(g^{\prime },u^{\prime },h^{\prime },w^{\prime },v^{\prime }\right)$ and broadcasts $GP^{\prime }$ to all members of the system.

➁ When GA receives the updated global public parameter $GP^{\prime }$, it runs algorithm $\mathrm{GA}.\mathrm{Setup}(GP^{\prime })$ to obtain $\left(GPK,GMK\right)$ and sends it to $W_{\mathrm{GA}}$, and $W_{\mathrm{GA}}$ performs the algorithm $W_{\mathrm{GA}}.\mathrm{GA}.\mathrm{Setup}$.

$W_{\mathrm{GA}}.\mathrm{GA}.\mathrm{Setup}(GP^{\prime },GPK,GMK)\to (GPK^{\prime },GMK^{\prime })$. $W_{\mathrm{GA}}$ randomly selects $f\leftarrow Z_{p}$, sets $\alpha^{\prime }=\alpha +f$, calculates and outputs $GPK^{\prime }=GPK\cdot e{\left(g^{\prime },g^{\prime }\right)}^{f}=e{\left(g^{\prime },g^{\prime }\right)}^{\alpha +f}=e{\left(g^{\prime },g^{\prime }\right)}^{\alpha^{\prime }}$, $GMK^{\prime }=GMK+f=\alpha +f=\alpha^{\prime }$, while keeping *f* by itself.

➂ After receiving the updated $GP^{\prime }$, AA runs $\mathrm{AA}.\mathrm{Setup}(GP,k,U_{k})\to (APK_{k},AMK_{k})$ and sends $\left(APK_{k},AMK_{k}\right)$ to $W_{\mathrm{AA}}$. $W_{\mathrm{AA}}$ performs the algorithm $W_{\mathrm{AA}}.\mathrm{Setup}$.

$W_{\mathrm{AA}}.\mathrm{Setup}(GP^{\prime },APK_{k},AMK_{k})$$\to (AP{K_{k}}^{\prime },AM{K_{k}}^{\prime })$. $W_{\mathrm{AA}}$ randomly selects ${\tilde{\alpha }}_{k}\leftarrow Z_{p}$, sets ${\alpha_{k}}^{\prime }=\alpha_{k}+{\tilde{\alpha }}_{k}$, $({\hat{u}}^{\prime },{\hat{h}}^{\prime })=\left({u^{\prime }}^{{\alpha_{k}}^{\prime }},{h^{\prime }}^{{\alpha_{k}}^{\prime }}\right)$ and ouputs $AP{K_{k}}^{\prime }=\left(\hat{u}\cdot u^{\prime {\tilde{\alpha }}_{k}},\hat{h}\cdot h^{\prime {\tilde{\alpha }}_{k}}\right)=$$\left({u^{\prime }}^{\alpha_{k}+{\tilde{\alpha }}_{k}},{h^{\prime }}^{\alpha_{k}+{\tilde{\alpha }}_{k}}\right)=\left({u^{\prime }}^{{\alpha_{k}}^{\prime }},{h^{\prime }}^{{\alpha_{k}}^{\prime }}\right)=({\hat{u}}^{\prime },{\hat{h}}^{\prime })$, while keeping $AM{K_{k}}^{\prime }=\alpha_{k}+{\tilde{\alpha }}_{k}={\alpha_{k}}^{\prime }$ by itself.

(2) **Encryption phase**. DO and $W_{\mathrm{DO}}$ encrypt plaintext offline and online.

The DO runs $\mathrm{Encrypt}.\mathrm{off}(GP^{\prime },GPK^{\prime },APK^{\prime })$ and $\mathrm{Encrypt}.\mathrm{on}(GP^{\prime },APK_{A},m,A,CT_{off})$ to obtain the ciphertext $CT_{A}$ of the message *m* under the access structure A, and sends $CT_{A}$ to $W_{\mathrm{DO}}$ before uploading $CT_{A}$ to the CSP. $W_{\mathrm{DO}}$ performs the following algorithms.

➃ $W_{\mathrm{DO}}.\mathrm{Encrypt}.\mathrm{off}(GP^{\prime },GMK^{\prime },APK^{\prime })\to IT$. $W_{\mathrm{DO}}$ randomly selects $\tilde{s}\leftarrow Z_{p}$, ${\tilde{t}}_{j}\leftarrow Z_{p}$, j∈[J], calculates $\hat{K}m=e{\left(g^{\prime },g^{\prime }\right)}^{\alpha^{\prime }\tilde{s}}$, ${\hat{C}}_{0}={g^{\prime }}^{\tilde{s}}$, ${\hat{C}}_{j,1}={{\hat{h}}^{\prime }}^{-{\tilde{t}}_{j}}$, ${\hat{C}}_{j,2}={g^{\prime }}^{{\tilde{t}}_{j}}$, ${\hat{C}}_{j,3}={v^{\prime }}^{{\tilde{t}}_{j}}$, and outputs $IT=(\tilde{s},\hat{K}m,{\hat{C}}_{0},{\left\{{\hat{C}}_{j,1},{\hat{C}}_{j,2},{\hat{C}}_{j,3}\right\}}_{j\in [J]})$.

➄ $W_{\mathrm{DO}}.\mathrm{Encrypt}.\mathrm{on}(GP^{\prime },IT,CT_{A})\to {CT_{A}}^{\prime }$. $W_{\mathrm{DO}}$ sets ${\vec{y}}^{\prime }={\left(\tilde{s},{y_{2}}^{\prime },\cdots ,{y_{n}}^{\prime }\right)}^{T}$, where ${y_{i}}^{\prime }\leftarrow Z_{p}$, i=1,⋯,n, ${\vec{\lambda }}^{\prime }={\left({\tilde{\lambda }}_{1},\cdots ,{\tilde{\lambda }}_{l}\right)}^{T}=M{\vec{\mathrm{y;}}}^{\prime }$, $s^{\prime }=s+\tilde{s}$, ${t_{j}}^{\prime }=t_{j}+{\tilde{t}}_{j},{\lambda_{j}}^{\prime }=\lambda_{j}+{\tilde{\lambda }}_{j}$. It calculates ${\hat{C}}^{\prime }=C\cdot \hat{K}m=me{\left(g^{\prime },g^{\prime }\right)}^{\alpha^{\prime }\left(s+\tilde{s}\right)}=me{\left(g^{\prime },g^{\prime }\right)}^{\alpha^{\prime }s^{\prime }}$, where $s^{\prime }=s+\tilde{s}$. Then, it sets ${\hat{C}}_{0}^{\prime }=C_{0}\cdot {\hat{C}}_{0}={g^{\prime }}^{\left(s+\tilde{s}\right)}={g^{\prime }}^{s^{\prime }}$, ${\hat{C}}_{j,1}^{\prime }=C_{j,1}\cdot {\hat{C}}_{j,1}\cdot {{\hat{u}}^{\prime }}^{-\rho \left(j\right){\tilde{t}}_{j}}={\left({{\hat{u}}^{\prime }}^{\rho (j)}{\hat{h}}^{\prime }\right)}^{-t_{j}}\cdot {{\hat{h}}^{\prime }}^{-{\tilde{t}}_{j}}\cdot {{\hat{u}}^{\prime }}^{-\rho \left(j\right){\tilde{t}}_{j}}={\left({{\hat{u}}^{\prime }}^{\rho (j)}{\hat{h}}^{\prime }\right)}^{-{t_{j}}^{\prime }}$, ${\hat{C}}_{j,2}^{\prime }=C_{j,2}\cdot {\hat{C}}_{j,2}={g^{\prime }}^{\left(t_{j}+{\tilde{t}}_{j}\right)}={g^{\prime }}^{{t_{j}}^{\prime }}$, ${\hat{C}}_{j,3}^{\prime }=C_{j,3}\cdot {\hat{C}}_{j,3}\cdot {w^{\prime }}^{{\tilde{\lambda }}_{j}}=({v^{\prime }}^{t_{j}}{w^{\prime }}^{\lambda_{j}})\cdot {v^{\prime }}^{{\tilde{t}}_{j}}{w^{\prime }}^{{\tilde{\lambda }}_{j}}={v^{\prime }}^{\left(t_{j}+{\tilde{t}}_{j}\right)}{w^{\prime }}^{\left(\lambda_{j}+{\tilde{\lambda }}_{j}\right)}={v^{\prime }}^{{t_{j}}^{\prime }}{w^{\prime }}^{{\lambda_{j}}^{\prime }}$, and outputs the updated ciphertext

$CT^{\prime }=\left(A,{\hat{C}}^{\prime },{{\hat{C}}_{0}}^{\prime },{\left\{{\hat{C}}_{j,1}^{\prime },{\hat{C}}_{j,2}^{\prime },{\hat{C}}_{j,3}^{\prime }\right\}}_{j\in \left[l\right]}\right)$.

(3) **Key generation phase**. GA, AA, $W_{\mathrm{GA}}$ and $W_{\mathrm{AA}}$ generate decryption key offline and online for user.

GA runs $\mathrm{GA}.\mathrm{KeyGen}.\mathrm{off}(GP^{\prime },GMK)\to ugsk$, and sends ugsk to $W_{\mathrm{GA}}$. ${AA}_{k}$ runs $\mathrm{AA}.\mathrm{KeyGen}.\mathrm{off}(GP^{\prime },AMK_{k})$ and $\mathrm{AA}.\mathrm{KeyGen}.\mathrm{on}(GP^{\prime },GID,APK_{k},S_{GID,k},uask_{off})$, then sends $uask_{on,S_{GID}}$ to $W_{\mathrm{AA}}$. $W_{\mathrm{GA}}$ and $W_{\mathrm{AA}}$ perform the following algorithms.

➅ $W_{\mathrm{GA}}.\mathrm{GAKeyGen}.\mathrm{off}\left(GP^{\prime },f\right)\to ISK$. $W_{\mathrm{GA}}$ randomly selects $\tilde{r}\leftarrow Z_{p}$, calculates ${\hat{K}}_{0}={g^{\prime }}^{f}{w^{\prime }}^{\tilde{r}}$, ${\hat{K}}_{3}={g^{\prime }}^{\tilde{r}}$, $\hat{D}={v^{\prime }}^{-\tilde{r}}$, and outputs an intermediate secret key $ISK=(\tilde{r},{\hat{K}}_{0},{\hat{K}}_{3},\hat{D})$.

➆ $W_{\mathrm{GA}}.\mathrm{GAKeyGen}.\mathrm{on}\left(GP^{\prime },ISK,ugsk\right)\to ugsk^{\prime }$. $W_{\mathrm{GA}}$ sets $r^{\prime }=\tilde{r}+r$. It calculates ${\hat{K}}_{0}^{\prime }={\hat{K}}_{0}\cdot K_{0}={g^{\prime }}^{\left(\alpha +f\right)}{w^{\prime }}^{\left(\tilde{r}+r\right)}={g^{\prime }}^{\alpha^{\prime }}{w^{\prime }}^{\left(\tilde{r}+r\right)}={g^{\prime }}^{\alpha^{\prime }}{w^{\prime }}^{r^{\prime }}$, ${\hat{K}}_{3}^{\prime }={\hat{K}}_{3}\cdot K_{3}={g^{\prime }}^{\left(r+\tilde{r}\right)}={g^{\prime }}^{r^{\prime }}$, ${\hat{D}}^{\prime }={v^{\prime }}^{-(r+\tilde{r})}={v^{\prime }}^{-r^{\prime }}$, outputs the updated $ugsk^{\prime }=\left({\hat{K}}_{0}^{\prime },{\hat{K}}_{3}^{\prime },{\hat{D}}^{\prime }\right)$ and sends it to the user.

➇ $W_{\mathrm{AA}}.\mathrm{KeyGen}.\mathrm{off}\left(GP^{\prime },AM{K_{k}}^{\prime }\right)\to uas{k_{off}}^{\prime }$. $W_{\mathrm{AA}}$ randomly selects ${\tilde{r}}_{i}\leftarrow Z_{p}$, $i\in \left[\left|S_{GID,k}\right|\right]$, calculates ${\hat{K}}_{i,1}={g^{\prime }}^{\frac{{\tilde{r}}_{i}}{{\alpha_{k}}^{\prime }}}$, ${\hat{K}}_{i,2}={h^{\prime }}^{{\tilde{r}}_{i}}$, and outputs $uas{k_{off}}^{\prime }=\left({\tilde{r}}_{i},{\hat{K}}_{i,1},{\hat{K}}_{i,2}\right)$.

➈ $W_{\mathrm{AA}}.\mathrm{KeyGen}.\mathrm{on}\left(GP^{\prime },{\tilde{r}}_{i},S_{GID},GID,uask_{on},S_{GID,k},uas{k_{off}}^{\prime }\right)\to uask_{on,S_{GID}}^{\prime }$. $W_{\mathrm{AA}}$ sets ${r_{i}}^{\prime }=r_{i}+{\tilde{r}}_{i}$, calculates ${\hat{K}}_{i,1}^{\prime }=K_{i,1}\cdot {\hat{K}}_{i,1}={g^{\prime }}^{\frac{r_{i}}{{\alpha_{k}}^{\prime }}}\cdot {g^{\prime }}^{\frac{{\tilde{r}}_{i}}{{\alpha_{k}}^{\prime }}}={g^{\prime }}^{\frac{\left(r_{i}+{\tilde{r}}_{i}\right)}{{\alpha_{k}}^{\prime }}}={g^{\prime }}^{\frac{{r_{i}}^{\prime }}{{\alpha_{k}}^{\prime }}}$, ${\hat{K}}_{i,2}^{\prime }=K_{i,2}\cdot {\hat{K}}_{i,2}\cdot {\left({u^{\prime }}^{attr_{i}}\right)}^{{\tilde{r}}_{i}}={\left({u^{\prime }}^{attr_{i}}h^{\prime }\right)}^{r_{i}}\cdot {h^{\prime }}^{{\tilde{r}}_{i}}\cdot {u^{\prime }}^{attr_{i}\times {\tilde{r}}_{i}}={\left({u^{\prime }}^{attr_{i}}h^{\prime }\right)}^{{r_{i}}^{\prime }}$, and outputs $uask_{on,S_{GID}}^{\prime }={\left\{{\hat{K}}_{i,1}^{\prime },{\hat{K}}_{i,2}^{\prime }\right\}}_{i\in \left[\left|S_{GID,k}\right|\right]}$.

(4) **Decryption phase**. The $W_{\mathrm{DU}}$ re-randomizes the conversion key. The CSP uses the re-randomized conversion key to perform outsourcing decryption. $W_{\mathrm{DU}}$ performs the decryption algorithm.

After obtaining $\left(TK,RK)\leftarrow \mathrm{KeyGen}.\mathrm{ran}(ugsk^{\prime },uask_{on,S_{GID}}^{\prime }\right)$, DU sends TK to $W_{\mathrm{DU}}$ for the algorithm $W_{DU}.\mathrm{TKUpdate}$.

➉ $W_{DU}.\mathrm{TKUpdate}\left(TK\right)\to \left(TK^{\prime },\beta \right)$. $W_{\mathrm{DU}}$ randomly selects $\beta \leftarrow Z_{p}$ and calculates ${\tilde{K}}_{0}^{\prime }={\tilde{K}}_{0}^{\frac{1}{\beta }}={g^{\prime }}^{\frac{\alpha^{\prime }}{\tau \beta }}{w^{\prime }}^{\frac{r^{\prime }}{\tau \beta }}$, ${\tilde{K}}_{3}^{\prime }={\tilde{K}}_{3}^{\frac{1}{\beta }}={g^{\prime }}^{\frac{r^{\prime }}{\tau \beta }}$, ${\tilde{D}}^{\prime }={\tilde{D}}^{\frac{1}{\beta }}={v^{\prime }}^{-\frac{r^{\prime }}{\tau \beta }}$. For $i\in [|u_{k}|],k\in \left[K\right]$, it computes ${\tilde{K}}_{i,1}^{\prime }={{\tilde{K}}_{i,1}}^{\frac{1}{\beta }}={g^{\prime }}^{\frac{{r_{i}}^{\prime }}{{\alpha_{k}}^{\prime }\tau \beta }}$, ${\tilde{K}}_{i,2}^{\prime }={{\tilde{K}}_{i,2}}^{\frac{1}{\beta }}={\left({u^{\prime }}^{attr_{i}}h^{\prime }\right)}^{\frac{{r_{i}}^{\prime }}{\tau \beta }}$, outputs the updated conversion key $TK^{\prime }=\left(SGID,{\tilde{K}}_{0}^{\prime },{\tilde{K}}_{3}^{\prime },{\tilde{D}}^{\prime },{\left\{{\tilde{K}}_{i,1}^{\prime },{\tilde{K}}_{i,2}^{\prime }\right\}}_{i\in [|u_{k}|]}\right)$, while keeping β by itself.

The CSP receives $TK^{\prime }$, runs $\mathrm{Decrypt}.\mathrm{out}(TK^{\prime },CT^{\prime })\to TCT$, and sends TCT to $W_{\mathrm{DU}}$. $W_{\mathrm{DU}}$ performs the algorithm $W_{DU}.\mathrm{Decrypt}\left(TCT,\beta \right)$.

⑪ $W_{DU}.\mathrm{Decrypt}\left(TCT,\beta \right)\to TCT^{\prime }$. $W_{\mathrm{DU}}$ calculates $B^{\beta }=e{\left(g^{\prime },g^{\prime }\right)}^{\frac{\alpha^{\prime }s^{\prime }}{\tau }}$ and outputs $TCT^{\prime }=\left(B^{\beta },{\hat{C}}^{\prime }\right)$.

After receiving $TCT^{\prime }=\left(B^{\beta },{\hat{C}}^{\prime }\right)$, DU runs $\mathrm{Decrypt}.\mathrm{user}(RK,TCT^{\prime })\to m$, and obtains the plaintext *m*.

### 4.3. Security Analysis

**Theorem 2.**  *The proposed OO-MA-CP-ABE-CRFs is CPA secure and CRFs for GA, AAs, DO and DU maintain functionally, weakly preserve security, and weakly resist exfiltration if the basic structure of OO-MA-CP-ABE in Section 4.1 is CPA secure.*

**Proof.**  We prove the security of our construction via the following parts. □

**FUNCTIONALITY MAINTAINING.**

If the user attribute set $S_{\mathrm{GID}}$ satisfies the access policy A, then there is

$$\begin{array}{c} T_{j}=e\left({\tilde{K}}_{i,1}^{\prime },{\hat{C}}_{j,1}^{\prime }\right)\cdot e\left({\tilde{K}}_{i,2}^{\prime }\cdot {\tilde{D}}^{\prime },{\hat{C}}_{j,2}^{\prime }\right)\cdot e\left({\tilde{K}}_{3}^{\prime },{\hat{C}}_{j,3}^{\prime }\right)\\ =e({g^{\prime }}^{\frac{{r_{i}}^{\prime }}{{\alpha_{k}}^{\prime }\tau \beta }},{\left({{\hat{u}}^{\prime }}^{\rho (j)}{\hat{h}}^{\prime }\right)}^{-{t_{j}}^{\prime }})\cdot e\left({\left({u^{\prime }}^{attr_{i}}h^{\prime }\right)}^{\frac{{r_{i}}^{\prime }}{\tau \beta }}\cdot {v^{\prime }}^{-\frac{r^{\prime }}{\tau \beta }},{g^{\prime }}^{{t_{j}}^{\prime }}\right)\cdot e\left({g^{\prime }}^{\frac{r^{\prime }}{\tau \beta }},{v^{\prime }}^{{t_{j}}^{\prime }}{w^{\prime }}^{{\lambda_{j}}^{\prime }}\right)\\ =e\left({g^{\prime }}^{\frac{{r_{i}}^{\prime }}{{\alpha_{k}}^{\prime }\tau \beta }},{\left({{u^{\prime }}^{{\alpha_{k}}^{\prime }}}^{\rho (j)}{h^{\prime }}^{{\alpha_{k}}^{\prime }}\right)}^{-{t_{j}}^{\prime }}\right)\cdot e\left({\left({u^{\prime }}^{attr_{i}}h^{\prime }\right)}^{\frac{{r_{i}}^{\prime }}{\tau \beta }}\cdot {v^{\prime }}^{-\frac{r^{\prime }}{\tau \beta }},{g^{\prime }}^{{t_{j}}^{\prime }}\right)\cdot e\left({g^{\prime }}^{\frac{r^{\prime }}{\tau \beta }},{v^{\prime }}^{{t_{j}}^{\prime }}{w^{\prime }}^{{\lambda_{j}}^{\prime }}\right)\\ =e\left({g^{\prime }}^{\frac{{r_{i}}^{\prime }}{{\alpha_{k}}^{\prime }\tau \beta }},{\left({u^{\prime }}^{\rho (j)}h^{\prime }\right)}^{-{\alpha_{k}}^{\prime }{t_{j}}^{\prime }}\right)\cdot e\left({\left({u^{\prime }}^{attr_{i}}h^{\prime }\right)}^{\frac{{r_{i}}^{\prime }}{\tau \beta }}\cdot {v^{\prime }}^{-\frac{r^{\prime }}{\tau \beta }},{g^{\prime }}^{{t_{j}}^{\prime }}\right)\cdot e\left({g^{\prime }}^{\frac{r^{\prime }}{\tau \beta }},{v^{\prime }}^{{t_{j}}^{\prime }}{w^{\prime }}^{{\lambda_{j}}^{\prime }}\right)\\ =e\left({g^{\prime }}^{\frac{{r_{i}}^{\prime }}{{\alpha_{k}}^{\prime }\tau \beta }},{\left({u^{\prime }}^{\rho (j)}h^{\prime }\right)}^{-{\alpha_{k}}^{\prime }{t_{j}}^{\prime }}\right)\cdot e\left({\left({u^{\prime }}^{attr_{i}}h^{\prime }\right)}^{\frac{{r_{i}}^{\prime }}{\tau \beta }}\cdot {v^{\prime }}^{-\frac{r^{\prime }}{\tau \beta }},{g^{\prime }}^{{t_{j}}^{\prime }}\right)\cdot e\left({g^{\prime }}^{\frac{r^{\prime }}{\tau \beta }},{v^{\prime }}^{{t_{j}}^{\prime }}{w^{\prime }}^{{\lambda_{j}}^{\prime }}\right)\\ =e{\left(g^{\prime },\left({u^{\prime }}^{\rho (j)}h^{\prime }\right)\right)}^{-\frac{{r_{i}}^{\prime }{t_{j}}^{\prime }}{\tau \beta }}\cdot e\left({g^{\prime }}^{{t_{j}}^{\prime }},{\left({u^{\prime }}^{attr_{i}}h^{\prime }\right)}^{\frac{{r_{i}}^{\prime }}{\tau \beta }}\right)\cdot e\left({g^{\prime }}^{{t_{j}}^{\prime }},{v^{\prime }}^{-\frac{r^{\prime }}{\tau \beta }}\right)\cdot e\left({g^{\prime }}^{\frac{r^{\prime }}{\tau \beta }},{v^{\prime }}^{{t_{j}}^{\prime }}{w^{\prime }}^{{\lambda_{j}}^{\prime }}\right)\\ =e{\left(g^{\prime },\left({u^{\prime }}^{\rho (j)}h^{\prime }\right)\right)}^{-\frac{{r_{i}}^{\prime }{t_{j}}^{\prime }}{\tau \beta }}\cdot e{\left(g^{\prime },\left({u^{\prime }}^{attr_{i}}h^{\prime }\right)\right)}^{\frac{{r_{i}}^{\prime }{t_{j}}^{\prime }}{\tau \beta }}\cdot e\left({g^{\prime }}^{{t_{j}}^{\prime }},{v^{\prime }}^{-\frac{r^{\prime }}{\tau \beta }}\right)\cdot e\left({g^{\prime }}^{\frac{r^{\prime }}{\tau \beta }},{v^{\prime }}^{{t_{j}}^{\prime }}{w^{\prime }}^{{\lambda_{j}}^{\prime }}\right)\\ =e\left({g^{\prime }}^{{t_{j}}^{\prime }},{v^{\prime }}^{-\frac{r^{\prime }}{\tau \beta }}\right)\cdot e\left({g^{\prime }}^{\frac{r^{\prime }}{\tau \beta }},{v^{\prime }}^{{t_{j}}^{\prime }}{w^{\prime }}^{{\lambda_{j}}^{\prime }}\right)\\ =e\left({g^{\prime }}^{{t_{j}}^{\prime }},{v^{\prime }}^{-\frac{r^{\prime }}{\tau \beta }}\right)\cdot e\left({g^{\prime }}^{\frac{r^{\prime }}{\tau \beta }},{v^{\prime }}^{{t_{j}}^{\prime }}\right)\cdot e\left({g^{\prime }}^{\frac{r^{\prime }}{\tau \beta }},{w^{\prime }}^{{\lambda_{j}}^{\prime }}\right)\\ =e({g^{\prime }}^{\frac{r^{\prime }}{\tau \beta }},{w^{\prime }}^{{\lambda_{j}}^{\prime }})\\ =e{\left(g^{\prime },w^{\prime }\right)}^{\frac{r^{\prime }{\lambda_{j}}^{\prime }}{\tau \beta }} \end{array}$$

so successfully calculate

$$\begin{array}{c} B=\frac{e({\tilde{K}}_{0}^{\prime },{\hat{C}}_{0}^{\prime })}{\prod_{j\in I}{T_{j}}^{w_{j}}}=\frac{e({g^{\prime }}^{\frac{\alpha^{\prime }}{\tau \beta }}{w^{\prime }}^{\frac{r^{\prime }}{\tau \beta }},{g^{\prime }}^{s^{\prime }})}{\prod_{j\in I}{e(g^{\prime },w^{\prime })}^{\frac{r^{\prime }{\lambda_{j}}^{\prime }}{\tau \beta }}}\\ =\frac{e({g^{\prime }}^{\frac{\alpha^{\prime }}{\tau \beta }}{w^{\prime }}^{\frac{r^{\prime }}{\tau \beta }},{g^{\prime }}^{s^{\prime }})}{e{\left(g^{\prime },w^{\prime }\right)}^{\frac{r^{\prime }s^{\prime }}{\tau \beta }}}=\frac{e\left({g^{\prime }}^{\frac{\alpha^{\prime }}{\tau \beta }},{g^{\prime }}^{s^{\prime }}\right)\cdot e\left({w^{\prime }}^{\frac{r^{\prime }}{\tau \beta }},{g^{\prime }}^{s^{\prime }}\right)}{e{\left(g^{\prime },w^{\prime }\right)}^{\frac{r^{\prime }s^{\prime }}{\tau \beta }}}\\ =e\left({g^{\prime }}^{\frac{\alpha^{\prime }}{\tau \beta }},{g^{\prime }}^{s^{\prime }}\right)=e{\left(g^{\prime },g^{\prime }\right)}^{\frac{\alpha^{\prime }s^{\prime }}{\tau \beta }}, \end{array}$$

and then compute $\frac{{\hat{C}}^{\prime }}{{\left(B^{\beta }\right)}^{\tau }}=\frac{e{\left(g^{\prime },g^{\prime }\right)}^{\alpha^{\prime }s^{\prime }}\cdot m}{{\left(e\left(g^{\prime },{g^{\prime }}^{\frac{\alpha^{\prime }s^{\prime }}{\tau }}\right)\right)}^{\tau }}$ to obtain the plaintext *m*.

**CPA-SECURE.**

For any tampered implementation on the GA, AA, DO and DU that maintains functionality, we prove the CPA security of the constructed OO-MA-CP-ABE-CRFs with tampered algorithms Global.$\mathrm{Setu}p^{*}$, $\mathrm{GASetu}p^{*}$, $\mathrm{AASetu}p^{*}$, $\mathrm{GAKeyGen}.\mathrm{of}f^{*}$, $\mathrm{GAKeyGen}.on^{*}$, $\mathrm{AAKeyGen}.\mathrm{of}f^{*}$, $\mathrm{AAKeyGen}.on^{*}$, $\mathrm{KeyGen}.\mathrm{ra}n^{*}$, $\mathrm{Encrypt}{.\mathrm{off}}^{*}$ and $\mathrm{Encrypt}.on^{*}$.

Assuming that adversary A can break the CPA security of the OO-MA-CP-ABE-CRFs scheme with a non-negligible advantage ϵ, we can construct a PPT simulator B to break the CPA security of the basic OO-MA-CP-ABE scheme with the same advantage ϵ. In the OO-MA-CP-ABE-CRFs scheme, simulator B plays the role of a challenger, interacting with adversary A. Let C be the challenger in the OO-MA-CP-ABE scheme.

**Initialization**: B receives the access structure $A^{*}$ and the functionality maintaining algorithms from A, and sends them to C.

**Setup**: B receives GP=(g,h,u,v,w), GPK and $APK_{k}=\left(\hat{u},\hat{h}\right)$ from the C, randomly selects $a,b,c,d,e,f,{\tilde{\alpha }}_{k}\leftarrow Z_{p}$, calculates $g^{\prime }=g^{a}$, $u^{\prime }=u^{b}$, $h^{\prime }=h^{c}$, $w^{\prime }=w^{d}$, $v^{\prime }=v^{e}$, $u^{\prime {\tilde{\alpha }}_{k}}$, $h^{\prime {\tilde{\alpha }}_{k}}$, let $GP^{\prime }=\left(g^{\prime },u^{\prime },h^{\prime },w^{\prime },v^{\prime }\right)$, $GPK^{\prime }=GPK\cdot e{\left(g^{\prime },g^{\prime }\right)}^{f}$, $AP{K_{k}}^{\prime }=\left(\hat{u}\cdot u^{\prime {\tilde{\alpha }}_{k}},\hat{h}\cdot h^{\prime {\tilde{\alpha }}_{k}}\right)$, and passes $GP^{\prime }$, $GPK^{\prime }$ and $AP{K_{k}}^{\prime }$ to A.

**Phase 1**: B receives the key query about *S* and GID from A, passes them to C and obtains $ugsk=(K_{0},K_{3},D)$, $uask_{S_{GID}}=\left\{K_{i,1},K_{i,2}\right\}$, TK. Then, B randomly selects $\tilde{r},{\tilde{r}}_{i},\beta \leftarrow Z_{p}$, $i\in \left[\left|S_{GID,k}\right|\right]$, calculates ${\hat{K}}_{0}={g^{\prime }}^{f}{w^{\prime }}^{\tilde{r}}$, ${\hat{K}}_{3}={g^{\prime }}^{\tilde{r}}$, $\hat{D}={v^{\prime }}^{-\tilde{r}}$, ${\hat{K}}_{i,1}={g^{\prime }}^{\frac{{\tilde{r}}_{i}}{{\alpha_{k}}^{\prime }}}$, ${\hat{K}}_{i,2}={h^{\prime }}^{{\tilde{r}}_{i}}$, lets $ugsk^{\prime }=\left({\hat{K}}_{0}^{\prime },{\hat{K}}_{3}^{\prime },{\hat{D}}^{\prime }\right)=\left({\hat{K}}_{0}\cdot K_{0},{\hat{K}}_{3}\cdot K_{3},\hat{D}\cdot D\right)$, $uask_{on,S_{GID}}^{\prime }=\left\{{\hat{K}}_{i,1}^{\prime },{\hat{K}}_{i,2}^{\prime }\right\}=\left({\hat{K}}_{i,1}^{\prime }=K_{i,1}\cdot {\hat{K}}_{i,1},{\hat{K}}_{i,2}^{\prime }=K_{i,2}\cdot {\hat{K}}_{i,2}\cdot {\left({u^{\prime }}^{attr_{i}}\right)}^{{\tilde{r}}_{i}}\right)$, $TK^{\prime }=TK^{\frac{1}{\beta }}$, and passes $\left(ugsk^{\prime },uask_{S_{GID}}^{\prime }\right)$, and $TK^{\prime }$ to A.

**Challenge**: B sends two equal-length plaintexts $m_{0}$, $m_{1}$ to C and receives a challenge ciphertext $CT_{A}=(C,C_{0},\left\{C_{j,1},C_{j,2},C_{j,3}{\}}_{j\in [l]}\right)$. Then, B randomly selects $\tilde{s}\leftarrow Z_{p}$, ${\tilde{t}}_{j}\leftarrow Z_{p}$, j∈[J],${y_{i}}^{\prime }\leftarrow Z_{p}$, i∈[n], calculates $\hat{K}m=e{\left(g^{\prime },g^{\prime }\right)}^{\alpha^{\prime }\tilde{s}}$, ${\hat{C}}_{0}={g^{\prime }}^{\tilde{s}}$, ${\hat{C}}_{j,1}={{\hat{h}}^{\prime }}^{-{\tilde{t}}_{j}}$, ${\hat{C}}_{j,2}={g^{\prime }}^{{\tilde{t}}_{j}}$, ${\hat{C}}_{j,3}={v^{\prime }}^{{\tilde{t}}_{j}}$, lets $CT^{\prime }=\left(A,{\hat{C}}^{\prime },{\hat{C}}_{0}^{\prime },{\left\{{\hat{C}}_{j,1}^{\prime },{\hat{C}}_{j,2}^{\prime },{\hat{C}}_{j,3}^{\prime }\right\}}_{j\in \left[l\right]}\right)$, where ${\hat{C}}^{\prime }=C\cdot \hat{K}m$, ${\hat{C}}_{0}^{\prime }=C_{0}\cdot {\hat{C}}_{0}$, ${\hat{C}}_{j,1}^{\prime }=C_{j,1}\cdot {\hat{C}}_{j,1}\cdot {{\hat{u}}^{\prime }}^{-\rho \left(j\right){\tilde{t}}_{j}}$, ${\hat{C}}_{j,2}^{\prime }=C_{j,2}\cdot {\hat{C}}_{j,2}$, ${\hat{C}}_{j,3}^{\prime }=C_{j,3}\cdot {\hat{C}}_{j,3}\cdot {w^{\prime }}^{{\tilde{\lambda }}_{j}}$, ${\vec{y}}^{\prime }={\left(\tilde{s},{y_{2}}^{\prime },\cdots ,{y_{n}}^{\prime }\right)}^{T}$, $\vec{\lambda^{\prime }}={\left({\tilde{\lambda }}_{1},\cdots ,{\tilde{\lambda }}_{l}\right)}^{T}=M{\vec{y}}^{\prime }$ and passes $CT_{b}^{\prime }$ to A.

**Phase 2**: The process is the same as Phase 1.

**Guess**: The adversary A outputs a guess $b^{\prime }\in \left\{0,1\right\}$ for *b*. Then, B outputs the same guess $b^{\prime }$. Thus, if A has advantage ϵ in the OO-MA-CP-ABE-CRFs experiment, then B breaks the OO-MA-CP-ABE scheme with the same probability ϵ.

It is also known from the Theorem 1 of [32] that if an adversary breaks the scheme of [32] with a non-negligible advantage ϵ, a simulator can be constructed to break the q−type assumption in G with the same advantage ϵ. Therefore, if A has advantage ϵ in breaking our OO-MA-CP-ABE-CRFs scheme, then a simulator can be constructed to break the q−type assumption in G with the same advantage ϵ.

**WEAK SECURITY PRESERVATION AND WEAK EXFILTRATION RESISTANCE.**

According to the CPA security of the OO-MA-CP-ABE-CRFs scheme, the CRFs $W_{\mathrm{GA}}$, $W_{\mathrm{AA}}$, $W_{\mathrm{DO}}$ and $W_{\mathrm{DU}}$ corresponding to GA, AA, DO and DU always maintain weak preserve security. On the other hand, the proof of the CPA security further demonstrates that reverse firewalls $W_{\mathrm{GA}}$, $W_{\mathrm{AA}}$, $W_{\mathrm{DO}}$ and $W_{\mathrm{DU}}$ have weak resist exfiltration.

Combining the above discussion, the proof is completed.

## 5. Performance Evaluations

In order to compare our scheme with other schemes, we conduct a detailed analysis of property and performance analysis.

### 5.1. Property Comparison

We choose schemes [26,32,34,35,36,37] to compare with our scheme. These schemes are CP-ABE schemes that support the LSSS access structure. It can be seen from Table 1 that the scheme [37] only supports online/offline encryption (OO Encrypt). The scheme [26] supports online/offline key generation(OO KeyGen), OO Encrypt and CRF. However, the schemes [26,37] are not multi-authority. The schemes [32,34,35,36] are multi-authority, but the scheme [32] only supports OO KeyGen and OO Encrypt without CRF. The scheme [36] also only supports OO Encrypt. Only our scheme meets all the above properties, so it is more suitable for IoT.

### 5.2. Performance Analysis

In order to simulate the time cost of computing operations, Table 2 lists the complexity analysis of system setup, key generation, encryption, and decryption. We define P as a bilinear pairing operation, E as an exponentiation operation, and M as a multiplication operation. We use *K*, *S*, *l* and *I* to represent the number of attribute authorities, the number of user attributes, the number of rows in the LSSS matrix, and the row set of the LSSS matrix used for decryption. We consider the time cost of user key generation, user encryption and user decryption. Although CRFs are added to our scheme, and CRFs also generate keys and perform encryption, the time cost of this part does not belong to users, so the additional cost caused by CRFs can be ignored. In addition, considering that the offline phase does not affect the actual cost of the online part of the user in the actual scenario, we only consider the time cost of the online phase in the efficiency analysis.

As shown in Table 3, we show the storage cost of our scheme and schemes [26,32,34,35,36], respectively, in the public parameters, ciphertext and user decryption key, where |G| is the number of elements in group G, |$G_{T}$| is the number of elements in group $G_{T}$. Because these schemes are CP-ABE schemes, the size of *l* is proportional to the size of the ciphertext, and the size of *S* is proportional to the size of the user decryption key. Compared to [36], our scheme has better storage cost for public parameters, ciphertext and user decryption key. Compared with [26,32,34,35], the storage cost of ciphertext and the user decryption key is approximately equal.

It is worth noting that we implement the simulation of the algorithm by deploying the Charm-Crypto cryptographic library based on the Python language development framework in the Ubuntu system. The experimental environment is Ubuntu 18.04.6, 12th Gen Intel(R) Core(TM) i7-12700H 4-core 2.30 GHz processor and 4 GB of RAM.

In the experiment, we imported the PBC module and selected the parameter value “SS512”, type A curve to generate the prime order bilinear group G. We further try to perform 1000 repeated experiments and take the average to estimate the running time of bilinear pairing operation, exponentiation operation and multiplication operation in G, respectively. The results show that the average time cost of the bilinear pairing operation is 2.05 ms, the average time cost of the exponentiation operation is 2.80 ms, and the average time cost of the multiplication operation is 2.82 ms. The source code can be obtained at https://github.com/abcde123411/OO-MA-CP-ABE-CRF (accessed on 2 April 2023. Finally, we present the results in Table 4.

**Figure 2:**
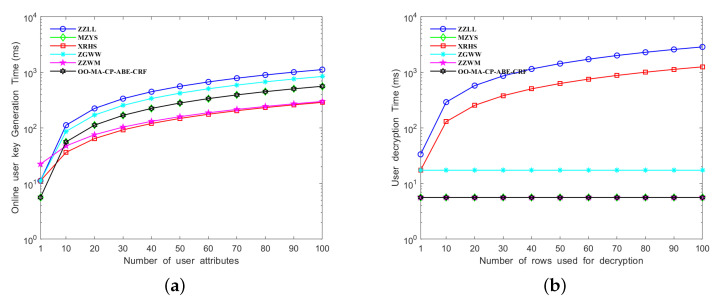
Computational cost comparison of online key generation and decryption: (**a**) The Online Key Generation Cost; (**b**) The Decryption Cost.

**Figure 3:**
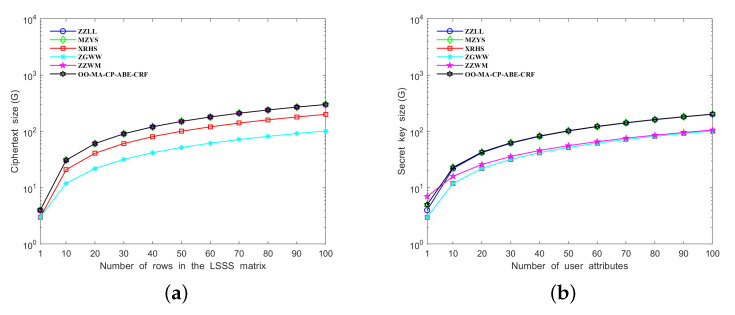
Storage cost comparison of ciphertext and secret key: (**a**) The Ciphertext Storage Cost; (**b**) The Secret Key Storage Cost.

In order to show the computational time cost and the storage cost of our scheme and other schemes, we make a comparison with Zhang et al. scheme [32] (ZZLL), Ma et al. scheme [26] (MZYS), Xie et al. scheme [34] (XRHS), Zhang et al. scheme [35] (ZGWW) and Zhang et al. scheme [36] (ZZWM). By analyzing the calculation overhead of the online user key generation phase, user decryption phase and the storage overhead of ciphertexts and keys in the system, there is a slight difference between the ZZWM scheme [36] and other schemes. In addition, we let the number of attribute authorities *K* be 1, the number of users *N* and the depth *d* is 0, $Z_{p}$ is equal to G to facilitate unified comparison, as shown in Figure 2 and Figure 3.

In Figure 2a, the curve of MZYS scheme [26] coincides with that of our scheme, which shows that the time cost of our scheme in the key generation phase is the same as that of MZYS scheme [26], which has obvious advantages over XRHS scheme [34] and ZZWM scheme [36]. In Figure 2b, the curve of MZYS scheme [26] coincides with that of our scheme. With the help of outsourced decryption technology, the time overhead of our scheme in the decryption phase is very considerable.

As can be seen from Table 3, since the ciphertext storage cost of each scheme contains a constant $G_{T}$, we can ignore it in the analysis of the storage cost of ciphertext. As shown in Figure 3a, the curves of MZYS scheme [26], ZZLL scheme [32] and ZZWM scheme [36] coincide with that of our scheme, which shows that the ciphertext storage cost of our scheme is equivalent to that of [26,32,36]. As shown in Figure 3b, the curves of XRHS scheme [34], ZGWW scheme [35] and ZZWM scheme [36] are coincident, and the curves of MZYS scheme [26] and ZZLL scheme [32] coincide with that of our scheme, which shows that the secret key storage cost of our scheme is equivalent to that MZYS scheme [26] and ZZLL scheme [32].

## 6. Real-World Application

**Figure 4:**
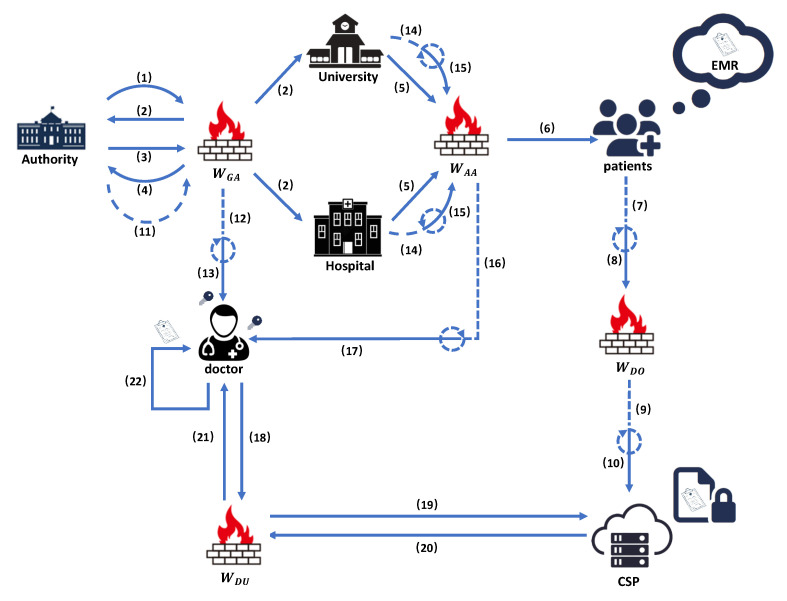
The OO-MA-CP-ABE-CRFs in an e-health system.

In this section, we will provide a detailed description of the practical application of our OO-MA-CP-ABE-CRFs scheme in the e-health system, as shown in Figure 4, which shows the true practical value of the scheme. The following steps are required.

(1) Patients and doctors need to register in the system. The superior management organization of the university and affiliated hospital executes algorithm Global.Setup to generate system global parameters and send them to registered users.

(2) Given that malicious adversaries may threaten the security of the system through backdoor attacks, the CRF corresponding to the superior management organization executes algorithm $W_{\mathrm{GA}}$.Global.Setup to randomize the system’s global parameters and broadcasts the updated results across the network.

(3) The superior management organization executes the algorithm GA.Setup to generate the public/private key pair.

(4) The CRF of the superior management organization runs the algorithm $W_{\mathrm{GA}}.\mathrm{GA}.\mathrm{Setup}$ to update the public/private key pair, and returns the results to the superior management organization.

(5) The school and hospital, as attribute authorities in the system, respectively, execute the algorithm AA.Setup to generate their own public/private key pair.

(6) To prevent this process from being compromised, the CRFs of the school and hospital execute the algorithm $W_{\mathrm{AA}}.\mathrm{Setup}$ to randomize their respective public and private key pair.

(7) Considering that most patients usually use resource-constrained mobile devices, some computational operations are performed in the Encrypt.off algorithm. This will ensure that mobile device resources are not excessively consumed when performing online encryption operations.

(8) Patient sets access control policies and executes the algorithm Encrypt.on to encrypt Electronic Medical Records (EMR).

(9) If the encryption process is compromised, the patient’s EMR may be compromised, directly endangering the privacy and security of the patient. To avoid this situation, the CRF of the patient executes the algorithm $W_{\mathrm{DO}}.\mathrm{Encrypt}.\mathrm{off}$ to generate intermediate ciphertext offline.

(10) The CRF of the patient executes the algorithm $W_{\mathrm{DO}}.\mathrm{Encrypt}.\mathrm{on}$ to update the ciphertext and stores the results in the CSP.

(11) The superior management organization executes the algorithm GA.KeyGen.off offline to generate a portion of the decryption key for registered users.

(12) The CRF of the superior management organization executes the algorithm

$W_{\mathrm{GA}}.\mathrm{GAKeyGen}.\mathrm{off}$ in the offline phase to generate an intermediate conversion key.

(13) The CRF of the superior management organization executes the algorithm

$W_{\mathrm{GA}}.\mathrm{GAKeyGen}.\mathrm{on}$ to generate a portion of the updated decryption key and sends the result to the doctor.

(14) School and hospital execute the algorithm AA.KeyGen.off in the offline phase to provide pre-computing services for the decryption key generation of doctors.

(15) The school and affiliated hospital execute the algorithm AA.KeyGen.on to generate the corresponding decryption key for the doctor based on their attribute set.

(16) Considering the backdoor attacks, the CRFs of the school and hospital execute the algorithm $W_{\mathrm{AA}}.\mathrm{KeyGen}.\mathrm{off}$ to update a portion of the decryption key offline.

(17) The CRF of the school and hospital execute the algorithm $W_{\mathrm{AA}}.\mathrm{KeyGen}.\mathrm{on}$ to update the doctor’s decryption key online and send the result to the doctor.

(18) Because we use outsourced decryption, the doctor executes the algorithm KeyGen.ran to generate a conversion key and a recovery key.

(19) If the conversion key is compromised, it may cause serious consequences. To this end, the CRF of the doctor executes the algorithm $W_{DU}.\mathrm{TKUpdate}$ to randomize the conversion key, and sends the updated result to the CSP.

(20) The CSP executes the algorithm Decrypt.out to pre-decrypt and obtain the transformed ciphertext.

(21) The doctor’s CRF executes the algorithm $W_{DU}.\mathrm{Decrypt}$ to partially decrypt transformed ciphertext.

(22) Doctors execute the algorithm Decrypt.user, and only authorized doctors can successfully obtain the patient’s EMR.

## 7. Conclusions

To solve the problem of data privacy security in IoT, we propose an OO-MA-CP-ABE-CRFs scheme. This scheme can not only protect the security of data but also achieve fine-grained access control of data. In addition, our scheme uses multi-authority technology to further reduce the trust of a single authority, uses CRF technology, which can effectively resist the ex-filtration of secret information, fully protect the privacy and security of users, and adopt online/offline and outsourcing decryption technology to reduce users’ storage and computing cost. The security proof and experimental comparison of the scheme show that our scheme is more suitable for data sharing for IoT.

Although the OO-MA-CP-ABE-CRFs scheme implements multi-authority, it requires a global identity authority. In future work, we will study how to remove the global identity authority so that each attribute authority has equal status. We can consider introducing a user’s identity or blockchain to achieve this. In addition, in order to make this scheme suitable for resource-constrained IOT devices, we need to further optimize the efficiency of the encryption algorithm. We can consider adopting techniques such as obfuscation encryption and reducing the size of ciphertext.

## Figures and Tables

**Table 1:** Comparison of properties.

Schemes	Multi-Authority	Online/Offline Key Generation	Online/Offline Encryption	CRF
[26]	×	✓	✓	✓
[37]	×	×	✓	×
[32]	✓	✓	✓	×
[34]	✓	×	×	×
[35]	✓	×	×	×
[36]	✓	×	✓	×
Proposed	✓	✓	✓	✓

The symbol ✓ represents that the scheme has this property, while the symbol × represents that the scheme does not have this property.

**Table 2:** Comparison of computational cost.

Schemes	System Setup	Online User Key Generation	Online User Encryption	User Decryption
[32]	1P+(2K+1)E	3|S|E+|S|M	1M	(3|I|+1)P+3|I|E+(5|I|+1)M
[26]	1P+7E	|S|E+|S|M	(2l+1)M +2lE	1E+1M
[34]	1P+3E	(|S|+2)E+1M	(1l+1)M+(3l+2)E	(2|I|+1)P+2|I|E+(1|I|+1)M
[35]	1P+(2K−1)E	(3|S|+1)E	lP+(l+3)E	3P+4E
[36]	(|U|+2N−1)E	(6K+S)E+KM	$\left(N^{\prime }+1\right)E+\left(l+1\right)M$	1E+1M
Proposed	1P+(4K+6)E+1M	|S|E+|S|M	(2l+1)M +2lE	1E+1M

**Table 3:** Comparison of storage cost.

Schemes	Public Parameters	Ciphertext	User Decryption Key
[32]	$\left(2K+5\right)|G|+|G_{T}|$	$\left(3l+1\right)|G|+|G_{T}|$	(2S+2)|G|
[26]	$5|G|+|G_{T}|$	$\left(3l+1\right)|G|+|G_{T}|$	(2S+3)|G|
[34]	$3|G|+|G_{T}|$	$\left(2l+1\right)|G|+|G_{T}|$	(S+2)|G|
[35]	$\left|G|+\right|G_{T}|$	$\left(l+2\right)|G|+|G_{T}|$	(S+2)|G|
[36]	$\left(2K+|U|+2N+2)|G|+K\right|G_{T}|$	$\left(3l+\frac{N}{2}+1\right)\left|G|+\right|G_{T}|$	$\left(\left(4+d\right)K+S\right)|G|+2K|Z_{p}|$
Proposed	$\left(2K+5\right)|G|+|G_{T}|$	$\left(3l+1\right)|G|+|G_{T}|$	(2S+3)|G|

**Table 4:** Time cost of sub-operations.

Operation	Time Cost
bilinear pairing operation	2.05 ms
exponentiation in G	2.80 ms
multiplication in G	2.82 ms

## Data Availability

Not applicable.

## Appendix A

**Definition A1.** 
***(Cryptographic Reverse Firewall (CRF).)** A cryptographic reverse firewall (CRF) is a stateful algorithm W that takes its state and message as input and outputs an updated state and message. For simplicity, we do not explicitly indicate the state information of W. For a party P and reverse firewall W in the system, we use W∘P to define the composed party. When a composed party W∘P engages in the protocol, the state of W is initialized as a public parameter. If W is meant to be composed with a party P, then we call it a reverse firewall for P. For a party P=(receive,next,output) and CRF W with an initial state of σ, we define a composed party W∘P,where $W\circ P:=(\mathrm{receiv}e_{W\circ P}\left(\sigma ,m\right)=\mathrm{receiv}e_{P}\left(\sigma ,W\left(m\right)\right)$, $\mathrm{nex}t_{W\circ P}\left(\sigma \right)=W\left(\mathrm{nex}t_{P}\left(\sigma \right)\right)$, $\mathrm{outpu}t_{W\circ P}\left(\sigma \right)=\mathrm{outpu}t_{P}\left(\sigma \right)$.*

**Definition A2.** 
***(Functionality-maintaining CRFs.)** For any CRF W and any party P, let $W^{1}\circ P=W\circ P$. Meanwhile, for k≥2, let $W^{k}\circ P=W\circ \left(W^{k-1}\circ P\right)$. For the scheme E that satisfies a certain functional requirement F, if the party P in the scheme E whose $W^{k}\circ P$ is bounded by any polynomial k≥1 always maintains F, then we say that the reverse firewall W maintains the normal function of all the parties P in the scheme E. When F, P, and E are clear, it is said that the reverse firewall W maintains functionality.*

**Definition A3.** 
***(Weakly security-preserving CRFs.)** For a scheme E that satisfies security requirement S and functionality F and a CRF W. If for any PPT adversary A maintaining F, the scheme $E_{P\to W\circ \hat{P}}$, where P is replaced by $W\circ \hat{P}$ in scheme E and $\hat{P}$ denote adversarial implementations during protocol function maintenance, satisfies the security requirement S, then CRF W can weakly preserve S of the party P in E. When E, P, S and F are clear, we simply say that W weakly preserves security.*

**Definition A4.** 
***(Weakly exfiltration-resistant CRFs.)** For a scheme E and reverse firewall W satisfying functional F, if any PPT adversary A whose advantage $Adv_{A,W}^{\mathrm{LEAK}}\left(l\right)=$$Pr\left[\mathrm{LEAK}(E,P_{1},P_{2},W,l)=1\right]-\frac{1}{2}$ in the security parameter l provided that $P_{1}^{*}$ maintains F for $P_{1}$ is negligible, then we say the party $P_{1}$ against the party $P_{2}$ in scheme E, where the game $\mathrm{LEAK}(E,P_{1},P_{2},W,l)$ is as follows: we define A as the adversary, $\sigma_{P_{2}^{*}}$ as the state of $P_{2}^{*}$, $T^{*}$ is the transcript of running scheme $\varepsilon_{P_{1}\to P^{*},P_{2}\to P_{2}^{*}}\left(I\right)$. The adversary first chooses $P_{1}^{*},P_{2}^{*},I$ according to the security parameter l, and then chooses $b\overset{\$}{⟵}\left\{0,1\right\}$ randomly. If b=1, the challenger lets $P^{*}\leftarrow W\circ P_{1}^{*}$, otherwise lets $P^{*}\leftarrow W\circ P_{1}$, and outputs $T^{*}\leftarrow \varepsilon_{P_{1}\to P^{*},P_{2}\to P_{2}^{*}}\left(I\right)$. At last, the adversary outputs the guess $b^{*}\leftarrow A\left(T^{*},\sigma_{P_{2}^{*}}\right)$ for b, and outputs 1 if and only if $b^{*}=b$.*

## Appendix B

Let *U* be the full set of attributes, and A=(M,ρ) be the monotonic access structure. The OO-MA-CP-ABE-CRFs for access structure space A=(M,ρ) consists of 22 algorithms:

(1) Global.Setup(λ,U)→GP. This algorithm is run by the global identity authority GA. It inputs the security parameter λ, the attributes set *U* and outputs the global public parameter GP.

(2) $W_{\mathrm{GA}}$.Global.$\mathrm{Setup}(GP)\to GP^{\prime }$. This algorithm is run by the cryptographic reverse firewall $W_{\mathrm{GA}}$. On the input of a public parameter GP, it outputs the updated global public parameter $GP^{\prime }$.

(3) $\mathrm{GA}.\mathrm{Setup}(GP^{\prime })\to \left(GPK,GMK\right)$. This algorithm is run by GA. On input the updated global public parameter $GP^{\prime }$, it outputs the public/private key pair (GPK,GMK) of GA.

(4) $W_{\mathrm{GA}}.\mathrm{GA}.\mathrm{Setup}(GP^{\prime },GPK,GMK)\to (GPK^{\prime },GMK^{\prime })$. This algorithm is run by the reverse firewall $W_{GA}$ for GA. On input $GP^{\prime }$, GPK and GMK, it outputs the updated public/private key pair $\left(GPK^{\prime },GMK^{\prime }\right)$, and returns it to GA.

(5) $\mathrm{AA}.\mathrm{Setup}(GP,k,U_{k})\to (APK_{k},AMK_{k})$. This algorithm is run by attribute authority ${AA}_{k}$. It inputs GP, *k*, $U_{k}$, where $U_{k}$ is the set of attributes managed by ${AA}_{k}$, $U=\cup_{i\in \left[K\right]}U_{k},U_{i}\cap U_{j}=\emptyset ,i\neq j$. Finally, it outputs the public/private key pair $(APK_{k},AMK_{k})$ of attribute authority ${AA}_{k}$.

(6) $W_{\mathrm{AA}}.\mathrm{Setup}(GP^{\prime },APK_{k},AMK_{k})\to (AP{K_{k}}^{\prime },AM{K_{k}}^{\prime })$. This algorithm is run by the reverse firewall $W_{\mathrm{AA}}$ of the attribute authority ${AA}_{k}$. It inputs $GP^{\prime }$, ${APK}_{k}$, ${AMK}_{k}$ and ouputs the updated parameters $AP{K_{k}}^{\prime }$, $AM{K_{k}}^{\prime }$.

(7) $\mathrm{Encrypt}.\mathrm{off}(GP^{\prime },GPK^{\prime },APK^{\prime })\to CT_{off}$. This algorithm is run offline by the data owner DO. Input $GP^{\prime }$, $GPK^{\prime }$, $APK^{\prime }$, and output offline ciphertext ${CT}_{off}$.

(8) $\mathrm{Encrypt}.\mathrm{on}(GP^{\prime },APK^{\prime },m,A,CT_{off})\to CT_{A}$. This algorithm is run online by the data owner DO. Input $GP^{\prime }$, $APK^{\prime }$, *m*, A, ${CT}_{off}$, and output the ciphertext $CT_{A}$ of *m* under the access structure A.

(9) $W_{\mathrm{DO}}.\mathrm{Encrypt}.\mathrm{off}(GP^{\prime },GMK^{\prime },APK^{\prime })\to IT$. This algorithm is run offline by the reverse firewall $W_{\mathrm{DO}}$ of the data owner DO. On input $GP^{\prime }$, $GMK^{\prime }$ and $APK^{\prime }$, it outputs the updated intermediate ciphertext IT.

(10) $W_{\mathrm{DO}}.\mathrm{Encrypt}.\mathrm{on}(GP^{\prime },IT,CT)\to CT^{\prime }$. This algorithm is run online by the reverse firewall $W_{\mathrm{DO}}$ of the data owner DO. On input $GP^{\prime }$, IT and CT, it outputs the updated ciphertext $CT^{\prime }$.

(11) $\mathrm{GAKeyGen}.\mathrm{off}(GP^{\prime },GMK^{\prime })\to ugsk$. This algorithm is run offline by GA, inputs $GP^{\prime }$, $GMK^{\prime }$ and outputs the user’s decryption key ugsk.

(12) $W_{\mathrm{GA}}.\mathrm{GAKeyGen}.\mathrm{off}\left(GP^{\prime },f\right)\to ISK$. This algorithm is run offline by the reverse firewall $W_{\mathrm{GA}}$ of GA. On input $GP^{\prime }$ and *f*, it outputs the intermediate secret key ISK.

(13) $W_{\mathrm{GA}}.\mathrm{GAKeyGen}.\mathrm{on}\left(GP^{\prime },ISK,ugsk\right)\to ugsk^{\prime }$. This algorithm is run online by the reverse firewall $W_{\mathrm{GA}}$ of GA. On input $GP^{\prime }$, ISK, ugsk, it outputs the user’s updated decryption key $ugsk^{\prime }$.

(14) $\mathrm{AA}.\mathrm{KeyGen}.\mathrm{off}\left(GP^{\prime },AMK_{k}\right)\to uask_{off}$. This algorithm runs offline by ${AA}_{k}$. Inputs $GP^{\prime }$, ${AMK}_{k}$, and outputs the user’s offline decryption key ${uask}_{off}$.

(15) $\mathrm{AA}.\mathrm{KeyGen}.\mathrm{on}\left(GP^{\prime },GID,APK_{k},S_{GID,k},uask_{off}\right)\to uask_{on,S_{GID}}$. This algorithm runs online by ${AA}_{k}$, inputs $GP^{\prime }$, GID, ${APK}_{k}$, $S_{GID,k}$, ${uask}_{off}$, and outputs the decryption key ${uask}_{on,S_{GID}}$ of the user GID.

(16) $W_{\mathrm{AA}}.\mathrm{KeyGen}.\mathrm{off}\left(GP^{\prime },AM{K_{k}}^{\prime }\right)\to uas{k_{off}}^{\prime }$. This algorithm is run offline by the reverse firewall $W_{\mathrm{AA}}$ of ${AA}_{k}$, input $GP^{\prime }$, $AM{K_{k}}^{\prime }$, and output the updated offline decryption key $uas{k_{off}}^{\prime }$ of the user.

(17) $W_{\mathrm{AA}}.\mathrm{KeyGen}.\mathrm{on}\left(GP^{\prime },{\tilde{r}}_{i},S_{GID},GID,uask_{on},S_{GID,k},uas{k_{off}}^{\prime }\right)\to uask_{on,S_{GID}}^{\prime }$. This algorithm is run online by the reverse firewall $W_{\mathrm{AA}}$ of $AA_{k}$. Input $GP^{\prime }$, ${\tilde{r}}_{i}$, $S_{GID}$, GID, $uask_{on}$, $S_{GID,k}$, $uas{k_{off}}^{\prime }$, and output the user’s updated online decryption key $uask_{on,S_{GID}}^{\prime }$.

(18) $\mathrm{KeyGen}.\mathrm{ran}\left(ugsk^{\prime },uask_{on,S_{GID}}^{\prime }\right)\to \left(TK,RK\right)$. This algorithm is run by DU. Input $ugsk^{\prime }$, $uask_{on,S_{GID}}^{\prime }$, and output a conversion key TK and a retrieval key RK.

(19) $W_{\mathrm{DU}}.\mathrm{TKUpdate}\left(TK\right)\to \left(TK^{\prime },\beta \right)$. This algorithm is run by the reverse firewall $W_{\mathrm{DU}}$ of DU, which inputs TK, outputs an updated conversion key $TK^{\prime }$ and a corresponding random β.

(20) $\mathrm{Decrypt}.\mathrm{out}(TK^{\prime },CT^{\prime })\to TCT$. This algorithm is run by CSP. Input $TK^{\prime }$, $CT^{\prime }$ and output the transformed ciphertext TCT.

(21) $W_{\mathrm{DU}}.\mathrm{Decrypt}\left(TCT,\beta \right)\to TCT^{\prime }$. This algorithm is run by the reverse firewall $W_{\mathrm{DU}}$ of DU, which inputs the transformed ciphertext TCT, a random β and outputs an updated transformed ciphertext $TCT^{\prime }$.

(22) $\mathrm{Decrypt}.\mathrm{user}(RK,TCT^{\prime })\to m$. This algorithm is run by the data user DU, which inputs a retrieval key RK, the updated transformed ciphertext $TCT^{\prime }$ and outputs the plaintext *m*.

**Correctness**: For the fixed universe description *U*, the security parameter λ, the access structure space A=(M,ρ) and the message *m*, the correctness property requires that for all Setup(λ,U)→GP, all $W_{\mathrm{GA}}$. Global.$\mathrm{Setup}(GP)\to GP^{\prime }$, all $\mathrm{GA}.\mathrm{Setup}(GP^{\prime })\to \left(GPK,GMK\right)$, all $\mathrm{GA}.\mathrm{Setup}(GP^{\prime })\to \left(GPK,GMK\right)$, all $W_{\mathrm{GA}}.\mathrm{GA}.\mathrm{Setup}(GP^{\prime },GPK,GMK)\to$$\left(GPK^{\prime },GMK^{\prime }\right)$, all $\mathrm{AA}.\mathrm{Setup}(GP,k,U_{k})$$\to (APK_{k},AMK_{k})$, all $W_{\mathrm{AA}}.\mathrm{Setup}(GP^{\prime },APK_{k},AMK_{k})\to (AP{K_{k}}^{\prime },AM{K_{k}}^{\prime })$, all $\mathrm{Encrypt}.\mathrm{off}(GP^{\prime },GPK^{\prime },APK^{\prime })$→$CT_{off}$, all Encrypt.on($GP^{\prime },APK^{\prime },m,A,CT_{off})\to CT_{A}$, all $W_{\mathrm{DO}}.\mathrm{Encrypt}.\mathrm{off}(GP^{\prime },GMK^{\prime },APK^{\prime })\to IT$, all $W_{\mathrm{DO}}.\mathrm{Encrypt}.\mathrm{on}(GP^{\prime },IT,CT)\to CT^{\prime }$, all $\mathrm{GAKeyGen}.\mathrm{off}(GP^{\prime },GMK^{\prime })\to ugsk$, all $W_{\mathrm{GA}}.\mathrm{GAKeyGen}.\mathrm{off}\left(GP^{\prime },f\right)\to ISK$, all $W_{\mathrm{GA}}.\mathrm{GAKeyGen}.\mathrm{on}\left(GP^{\prime },ISK,ugsk\right)\to ugsk^{\prime }$, all $\mathrm{AA}.\mathrm{KeyGen}.\mathrm{off}\left(GP^{\prime },AMK_{k}\right)\to uask_{off}$, all $\mathrm{AA}.\mathrm{KeyGen}.\mathrm{on}\left(GP^{\prime },GID,APK_{k},S_{GID,k},uask_{off}\right)\to uask_{on,S_{GID}}$, all $W_{\mathrm{AA}}.\mathrm{KeyGen}.\mathrm{off}$$\left(GP^{\prime },AM{K_{k}}^{\prime }\right)\to uas{k_{off}}^{\prime }$, all $W_{\mathrm{AA}}.\mathrm{KeyGen}.\mathrm{on}\left(GP^{\prime },{\tilde{r}}_{i},S_{GID},GID,uask_{on},S_{GID,k},uas{k_{off}}^{\prime }\right)$$\to uask_{on,S_{GID}}^{\prime }$, all $\mathrm{KeyGen}.\mathrm{ran}\left(ugsk^{\prime },uask_{on,S_{GID}}^{\prime }\right)\to \left(TK,RK\right)$, all $W_{\mathrm{DC}}.\mathrm{TKUpdate}\left(TK\right)\to \left(TK^{\prime },\beta \right)$, all $\mathrm{Decrypt}.\mathrm{out}(TK^{\prime },CT^{\prime })\to TCT$, all $W_{\mathrm{DU}}.\mathrm{Decrypt}\left(TCT,\beta \right)\to TCT^{\prime }$. If *S* satisfies (M,ρ), then $\mathrm{Decrypt}.\mathrm{user}(RK,TCT^{\prime })\to m$.
